# Biofunctionalization of Collagen Barrier Membranes with Bone-Conditioned Medium, as a Natural Source of Growth Factors, Enhances Osteoblastic Cell Behavior

**DOI:** 10.3390/ijms26041610

**Published:** 2025-02-13

**Authors:** Harshitha Ashoka Sreeja, Emilio Couso-Queiruga, Clemens Raabe, Vivianne Chappuis, Maria B. Asparuhova

**Affiliations:** 1 Laboratory of Oral Cell Biology, Dental Research Center, School of Dental Medicine, University of Bern, Freiburgstrasse 3, 3010 Bern, Switzerland; 2 Department of Oral Surgery and Stomatology, School of Dental Medicine, University of Bern, Freiburgstrasse 7, 3010 Bern, Switzerland

**Keywords:** biomaterials, collagen, dental implants, bone augmentation, autologous bone, osteogenesis, growth factors, gene expression, cell growth, cell migration

## Abstract

A key principle of guided bone regeneration (GBR) is the use of a barrier membrane to prevent cells from non-osteogenic tissues from interfering with bone regeneration in patients with hard tissue deficiencies. The aim of the study was to investigate whether the osteoinductive properties of bone-conditioned medium (BCM) obtained from cortical bone chips harvested at the surgical site can be transferred to a native bilayer collagen membrane (nbCM). BCM extracted within 20 or 40 min, which corresponds to a typical implant surgical procedure, and BCM extracted within 24 h, which corresponds to BCM released from the autologous bone chips in situ, contained significant and comparable amounts of TGF-β1, IGF-1, FGF-2, VEGF-A, and IL-11. Significant (*p* < 0.001) quantities of BMP-2 were only detected in the 24-h BCM preparation. The bioactive substances contained in the BCM were adsorbed to the nbCMs with almost 100% efficiency. A fast but sequential release of all investigated proteins occurred within 6–72 h, reflecting their stepwise involvement in the natural regeneration process. BCM-coated nbCM significantly (*p* < 0.05) increased the migratory, adhesive, and proliferative capacity of primary human bone-derived cells (hBC), primary human periodontal ligament cells (hPDLC), and an osteosarcoma-derived osteoblastic cell line (MG-63) compared to cells cultured on BCM-free nbCM. The high proliferative rates of MG-63 cells cultured on BCM-free nbCM were not further potentiated by BCM, indicating that BCM-coated nbCM has no detrimental effects on cancer cell growth. BCM-coated nbCM caused significant (*p* < 0.05) induction of early osteogenic marker gene expression and alkaline phosphatase activity, suggesting an important role of BCM-functionalized nbCM in the initiation of osteogenesis. The 24-h BCM loaded on the nbCM was the only BCM preparation that caused significant induction of late osteogenic marker gene expression. Altogether, our data define the pre-activation of collagen membranes with short-term-extracted BCM as a potential superior modality for treating hard tissue deficiencies via GBR.

## 1. Introduction

It has been reported that bone augmentation is required for optimal implant placement in more than 50% of implant patients [1,2,3]. Guided bone regeneration (GBR) is a widely used and predictable technique for vertical and lateral bone augmentation of atrophic ridges, performed before or at the time of implant placement [4]. A key biological principle of GBR is the use of a barrier membrane [5,6] to establish the necessary protective space to support the intricate process of new bone formation and regeneration, which requires a high degree of cellular differentiation while protecting the bone graft placed in the defect area from the rapid ingrowth of connective and epithelial tissues. Native collagen membranes [6,7,8,9] have shown consistent results in a number of clinical studies [10,11,12,13,14,15,16]. Among the 28 types of collagens described, collagen type I and type III derived from porcine tissues are the most common components of currently available xenogenic collagen membranes. The membranes vary in thickness and porosity and consist of either a homogeneous collagen structure or a bilayered structure with a cell-occlusive outer layer that prevents migration of gingival fibroblasts and epithelial cells in the bone defect and a porous inner layer of collagen fiber bundles that facilitates tissue integration [17].

As a principal component of the connective tissue, collagen appears to be fully biocompatible [18], causes low immunogenicity [19], can stabilize clot formation by providing hemostatic wound coverage [20], attracts cells of different phenotypes, and binds various proteins naturally present in the surrounding fluids and tissues or produced de novo by cells attracted to the defect site [21]. While providing certain mechanical stability [22], pure collagen possesses no osteoinductive potential that is required to speed up the healing of bone defects in clinical practice [23]. Incomplete healing of alveolar bone defects after tooth extraction [24], which may compromise subsequent regenerative efforts, as well as poor osseointegration of dental implants despite prior bone augmentation [25,26], are commonly seen in dental practice. The clinical limitation of inefficient volume stability caused by the unpredictable degradation time and rate of commercially available unmodified collagen scaffolds can potentially be avoided by accelerating the bone formation process at the defect site. For this purpose, biofunctionalization of the collagen scaffolds is a viable option. Enhanced bone formation has been reported in studies investigating the binding of platelet-derived growth factor (PDGF) [27], bone morphogenetic proteins (BMP) [28,29,30], or the combination of fibroblast growth factor-2 (FGF-2) and stromal cell-derived factor-1α (SDF-1α) to collagen membranes [31]. A clinical study demonstrated a sustained release of PDGF-BB loaded onto a collagen membrane at sites of intraosseous defects augmented with β-tricalcium phosphate (β-TCP) [27]. Recombinant human BMP-2 loaded on a hydroxyapatite/β-TCP/collagen composite exhibited strong osteogenic potential that contributed to the successful repair of critical sized peri-implant defects in a canine model [30]. Another osteogenic member of the BMP family, namely BMP-9, loaded onto collagen membranes in a rabbit calvaria defect model resulted in favorable horizontal bone defect closure [28]. Furthermore, Xu et al. observed that the combination of FGF-2, SDF-1α, and a collagen scaffold enhanced bone marrow stem cell-induced periodontal ligament reconstruction, thereby promoting the survival of reimplanted teeth in beagle dogs [31].

In a recent study, we demonstrated that four commercially available porcine-derived collagen matrices can be efficiently loaded with enamel matrix derivative (EMD) or recombinant growth factors such as PDGF-BB, FGF-2, BMP-2, the transforming growth factor-β1 (TGF-β1), or growth and differentiation factor-5 (GDF-5) [32]. Furthermore, we observed preserved biological activity of EMD and recombinant BMP-2 adsorbed and released from each of the collagen biomaterials studied, as well as a clear stimulatory effect of each of the two substances on the osteogenic differentiation of osteoprogenitor cells grown on the scaffolds [33]. In terms of potential clinical application, the use of recombinant growth factors is limited by their short half-lives, necessitating the use of supraphysiologic doses that may raise toxicity concerns. However, the use of natural sources of growth factors containing physiological amounts of them for the biofunctionalization of collagen membranes, with the specific aim of acquiring osteoinductive potential and thus inducing bone formation in the GBR procedure, has not been systematically investigated. Bone-conditioned medium (BCM), which is obtained from intraorally harvested autologous bone chips and stored in a mixture of autologous blood and saline for the duration of the surgical site preparation [34], is a natural source of growth factors whose mechanism of action has been investigated in a number of studies [35,36,37,38,39,40,41,42,43]. We have shown that the technique by which the autologous bone chips are harvested [44] and the time frame for the BCM extraction [41] affect the BCM composition and the behavior of the cells treated with it. Furthermore, we have demonstrated a significant synergistic effect between BCM-coated deproteinized bovine bone mineral (DBBM) and freshly harvested bone chips on the osteogenic differentiation of bone-related cell cultures [45]. These findings have provided evidence for the strong osteoinductive potential of the BCM and convincingly justified a clinical approach combining autologous bone, BCM, and a second osteoconductive bone filler. The need to functionalize the biomaterials used in GBR arises from the known shortcomings of the two most commonly used bone fillers: (1) despite their osteoinductive, osteogenic, and osteoconductive properties, autologous bone graft harvesting is often associated with insufficient quantity and increased morbidity [46], and (2) the use of allografts or xenografts is associated with low or missing osteoinductivity as well as increased immunogenicity [47]. Furthermore, the increased adoption of immediate implant placement protocols in daily clinical practice contributes to increasing demands for fast healing and fast implant osseointegration [48]. Therefore, the aim of the present study was to gain further insight into the osteoinductive potential of BCM and its capacity to functionalize native collagen barrier membranes used in GBR. We quantitatively investigated the adsorption capacity of a native (non-crosslinked) bilayer collagen membrane (nbCM) for growth factors present in BCM and the subsequent growth factor release over a 4-day period. We hypothesized that clinically relevant short-term coating of the nbCM with short-term-extracted BCM will transfer the osteoinductive properties of the autograft to the membrane, leading to enhanced proliferative and osteogenic properties of primary human bone-derived cells (hBC), primary human periodontal ligament cells (hPDLC), and an osteosarcoma-derived osteoblastic cell line (MG-63) [49]. Thus, we also aimed to decipher a potential adverse effect of the BCM-coated nbCM on cancer cell behavior.

## 2. Results

### 2.1. Analysis of the Adsorption and Release of Growth Factors and Cytokines Involved in Bone Metabolism from BCM-Coated nbCM

**Figure 1:**
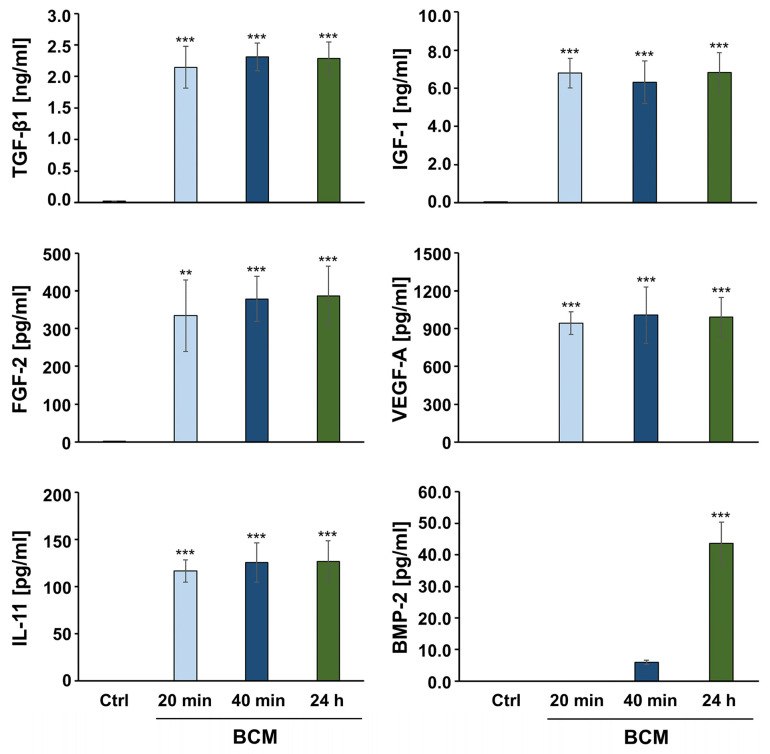
Release of various growth factors and cytokines involved in bone metabolism from cortical bone. Enzyme-linked immunosorbent assay (ELISA) quantification of TGF-β1, IGF-1, FGF-2, VEGF-A, IL-11, and BMP-2 proteins contained in bone-conditioned medium (BCM) extracted from cortical bone chips with Ringer’s solution (RS). BCM was collected at 20 min, 40 min, and 24 h. RS not containing bone particles represents the control (Ctrl) and exhibits no detectable levels of the proteins tested. Means ± SD from four independent BCM preparations from each type and significant differences to the control, *** *p* < 0.001, ** *p* < 0.01, are shown.

To analyze the adsorption rate and release kinetics of growth factors and cytokines from BCM-coated nbCM, we first determined the quantities of the respective bioactive substances in BCM prepared from cortical bone chips immersed in Ringer’s solution (RS) for the duration of 20 min, 40 min, or 24 h. The first two time periods for BCM harvesting correspond approximately to the time of a typical surgical procedure, whereas the 24-h BCM preparation (24 h BCM) corresponds to the BCM released from the autologous bone chips in situ, 24 h post-augmentation. Along with TGF-β1 and BMP-2, whose content in BCM was previously examined [41], we investigated a few other bioactive molecules with a well-known role in bone metabolism [50]. These are the insulin-like growth factor-1 (IGF-1), FGF-2, vascular endothelial growth factor-A (VEGF-A), and interleukin-11 (IL-11). Except for BMP-2, the quantities of TGF-β1, IGF-1, FGF-2, VEGF-A, and IL-11 did not significantly differ between the three BCM preparations, meaning that no significantly higher quantities of the growth factors were released from the bone chips after 20 min (Figure 1). The average concentrations measured in the BCM preparations amounted to 2.3 ± 0.3 ng/mL for TGF-β1, 6.7 ± 1.0 ng/mL for IGF-1, 366.4 ± 77.8 pg/mL for FGF-2, 980.9 ± 155.8 pg/mL for VEGF-A, and 122.8 ± 18.0 pg/mL for IL-11. In contrast, BMP-2 could not be detected in BCM prepared within 20 min, nor significant quantities (5.9 ± 0.6 pg/mL) of the growth factor, compared to the RS control, could be detected in the 40-min BCM preparation (40 min BCM). However, significantly higher (*p* < 0.001) amounts of BMP-2 equal to 43.6 ± 6.8 pg/mL were released from the bone chips in the 24 h BCM (Figure 1).

**Figure 2:**
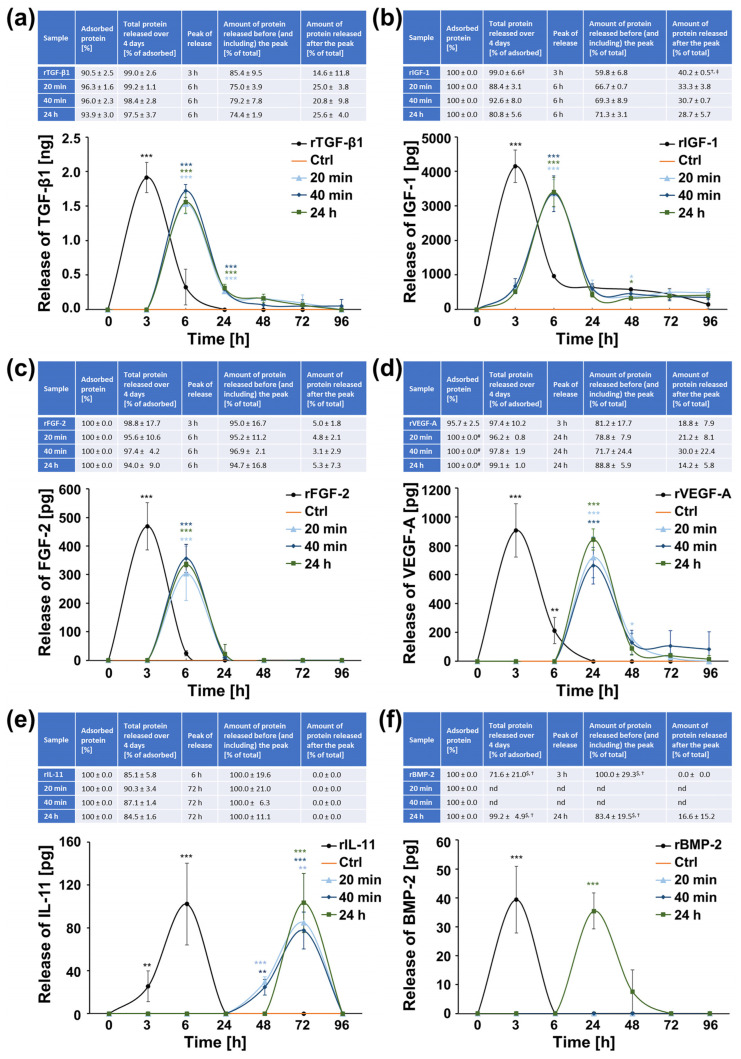
Adsorption and release of TGF-β1 (**a**), IGF-1 (**b**), FGF-2 (**c**), VEGF-A (**d**), IL-11 (**e**), and BMP-2 (**f**) from nbCM coated with either the respective recombinant (r) protein or BCM preparation. The nbCM was incubated for 10 min at room temperature in either RS containing the respective recombinant protein at the average concentration measured in the BCM preparations (cf. Figure 1) or each of the three BCM (20 min, 40 min, and 24 h). Hydration of the nbCM with RS was used as a control (Ctrl). Protein quantifications were performed by using colorimetric ELISA assays. Tables (a–f) represent: (1) quantifications of adsorbed protein (in percent); (2) total protein release (expressed as percent of adsorbed protein) from nbCM for a 4-day period; (3) the time point at which the highest protein release was observed (peak of release); (4) ELISA quantifications of the protein released before (and including) the peak expressed as percent of the total protein release for the entire test period (taken as 100%); (5) ELISA quantifications of the protein released after the peak until day 4 and expressed as in (4). Means ± SD from three independent experiments and significant differences (*p* < 0.05) between the experimental groups are shown. Significance was indicated with the following symbols: # denotes significantly higher than recombinant protein; $ denotes significantly higher than 20 min BCM; † denotes significantly higher than 40 min BCM; ‡ denotes significantly higher than 24 h BCM. Graphs represent the results from ELISA quantifications of the proteins measured in conditioned RS collected from the nbCM at the indicated time points over a 4-day period. Data represent means ± SD from three independent experiments. Significant differences between experimental groups, *** *p* < 0.001, ** *p* < 0.01, * *p* < 0.05.

Using the above-measured average concentrations of growth factors detected in the different BCM preparations, we coated nbCM with each of the recombinant (r) proteins or BCM preparations for a short, clinically relevant 10-min period. Hydration of nbCM in RS served as a control. Analyses of the adsorption rate to nbCM have indicated an efficiency greater than 90% for the adsorption of rTGF-β1 as well as TGF-β1 contained in each of the BCM preparations (Figure 2a). The nbCM adsorbed IGF-1, FGF-2, IL-11, and BMP-2, either as recombinant proteins or as part of the complex BCM protein mixture, with 100% efficiency (Figure 2b,c,e,f). Interestingly, the VEGF-A contained in each of the three BCM preparations was adsorbed with 100% efficiency, which appeared significantly (*p* < 0.05) higher compared to the rVEGF-A showing an adsorption rate of 96% (Figure 2d). The total protein release from nbCM, expressed as a percent of the adsorbed protein, for the test period of 4 days was extremely high, ranging from 72% for the rBMP-2 (Figure 2f) to 99% for the majority of the remaining proteins (Figure 2), including the BMP-2 as a constituent of the 24 h BCM (Figure 2f). In contrast to the BMP-2, rIGF-1 exhibited significantly (*p* < 0.05) higher release of 99% compared to the release of IGF-1 as a constituent of the 24 h BCM amounting to no more than 81% release over 4 days (Figure 2b).

As a next step, the release kinetics of the investigated factors were determined. Recombinant proteins released from nbCM were characterized with a burst release at 3 h for TGF-β1, IGF-1, FGF-2, VEGF-A, and BMP-2 and 6 h for IL-11. In contrast, growth factors released from the BCM-coated nbCM were characterized by a slightly delayed start of the release peaking at 6 h for TGF-β1, IGF-1, and FGF-2, at 24 h for VEGF-A and BMP-2, and at 72 h for IL-11 (Figure 2). A high amount of protein was released from both recombinant protein-coated and BCM-coated membranes, ranging from 72% to 97% of the total protein for TGF-β1, FGF-2, VEGF-A, and BMP-2 (Figure 2a,c,d,f). Therefore, only small to intermediate amounts of these growth factors, ranging from 3% to 30% of the total protein, were released after the initial burst phase and up to 4 days. Interestingly, whereas most of the proteins were characterized by a quick drop in the release following the peak, IGF-1 released from recombinant protein-coated nbCM within 3 h amounted to only 59.8% of the total protein, leading to a significantly (*p* < 0.05) higher amount of the growth factor reaching 40.2 ± 0.5%, which was released gradually after the peak compared to the IGF-1 release from BCM-coated nbCM (Figure 2b). In addition, IL-11 released from nbCM coated with the 20-min BCM preparation (20 min BCM) or the 40 min BCM was the only cytokine showing a gradual and very significant (*p* < 0.01) release at 48 h compared to the release from rIL-11-coated nbCM and before reaching a peak at 72 h (Figure 2e).

In summary, the relatively small quantities of cytokines and growth factors contained in BCM prepared within clinically relevant time periods can be efficiently and almost completely adsorbed on nbCM. The high overall protein release from nbCM over 4 days suggests that the highest biological activity of BCM or recombinant proteins used to coat a barrier membrane at physiological concentrations can be expected no later than 4 days post-augmentation. Among the bioactive substances studied, IL-11 was the only cytokine exhibiting a gradual release from nbCM coated with 20 or 40 min BCM, followed by a burst release that occurred later than 24 h post-coating.

### 2.2. Increased Migration Capacity of Three Osteoblast-like Cell Types Toward BCM-Coated nbCM

In a series of experiments, we analyzed the effects of BCM-coated nbCM on the behavior of three cell lines—primary hBCs and hPDLCs, as well as the immortal MG-63 cell line. The differentiation status of the MG-63 line and primary cells originating from different donors was characterized by analyzing the expression of early, intermediate, and late differentiation marker genes (Figure S1). The results showed that the primary hBCs and hPDLCs could be defined as pre-osteoblastic, while the MG-63 cells showed characteristics of mature osteoblasts. However, none of the cell lines expressed sclerostin, a marker for mature osteocytes. Therefore, we refer to all three cell lines with the general term “osteoblast-like cells” throughout the study.

**Figure 3:**
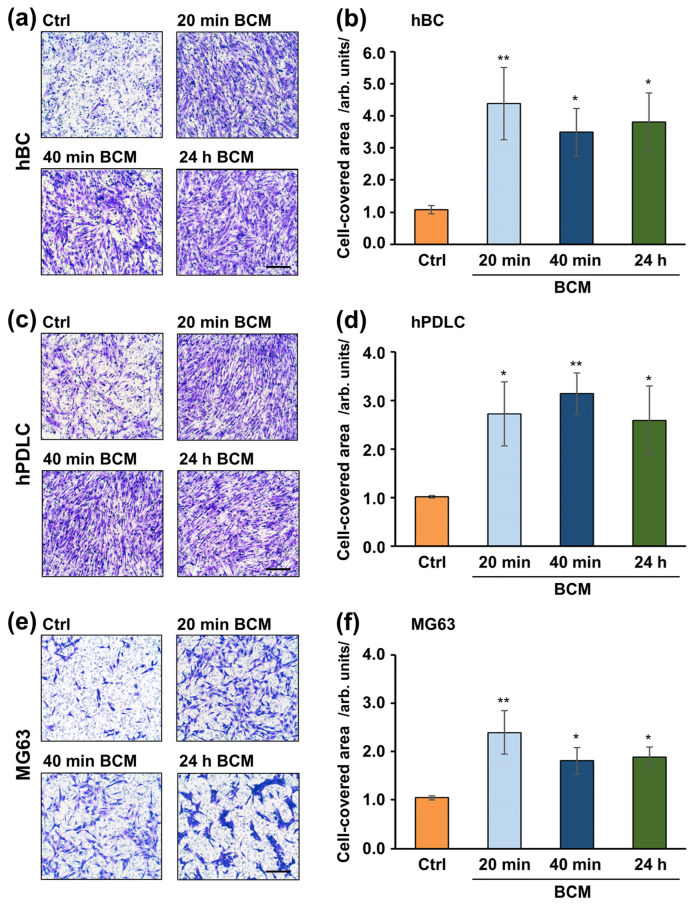
Induced migration capacity of osteoblast-like cell lines toward BCM-coated nbCM. Migration of primary human bone-derived cells (hBC) (**a**,**b**), primary human periodontal ligament cells (hPDLC) (**c**,**d**), and an osteosarcoma-derived immortal cell line (MG-63) (**e**,**f**) toward nbCMs coated with different BCM preparations (20 min, 40 min, or 24 h) was evaluated by a transwell migration assay utilizing ThinCert^®^ transwell PET membrane supports with 8 μm pore size. nbCM hydrated with RS was used as a control (Ctrl). (**a**,**c**,**e**) Representative images of fixed and stained cells that have migrated to the lower side of the filter in each of the experimental groups. Scale bar, 500 μm. (**b**,**d**,**f**) Quantification of the cell migration using the Image J software (version 1.50) measuring the area on the lower side of the filter covered with migrated cells. Data represent means ± SD from four independent experiments performed with (1) two independent BCM preparations, each used with two different cell donors for each of the two primary cell types, hBC and hPDLC, and (2) four independent BCM preparations used with the MG-63 cell line. Significant differences to the control, ** *p* < 0.01, * *p* < 0.05.

The migratory potential of hBCs, hPDLCs, and MG-63 cells toward nbCM coated with different BCM preparations was examined by using a transwell migration assay. Each of the three BCM preparations extracted within 20 min, 40 min, or 24 h caused a significant (*p* < 0.05) induction in the migration rate of each of the three cell types compared to the respective control cells, where the migration occurred in the presence of BCM-free nbCM hydrated with RS (Figure 3). No significant differences in the migration capacity induced by nbCMs coated with different BCM preparations were observed in any of the cell lines. Compared to hBCs and hPDLCs, MG-63 cells were significantly but least attracted to the BCM-coated nbCM, with no more than 2.4-fold higher potency than control cells (Figure 3e,f). The latter observation rules out the possibility that cancer cells, potentially present nearby the augmented site, could be attracted to the membrane compartment by the BCM.

### 2.3. Increased Expression of Adhesive Marker Genes in Three Osteoblast-like Cell Types Grown on BCM-Coated nbCM

It is becoming increasingly clear that the cellular interaction with implantable devices, such as collagen membranes used in GBR, and the induction of cell adhesion plays a central role in the expansion of osteoprogenitors and their subsequent differentiation [51]. Therefore, we performed a screen for the expression of several adhesive marker genes in hBCs, hPDLCs, and MG-63 cells seeded either on control nbCMs or nbCMs coated with the 20 min, 40 min, or 24 h BCM.

**Figure 4:**
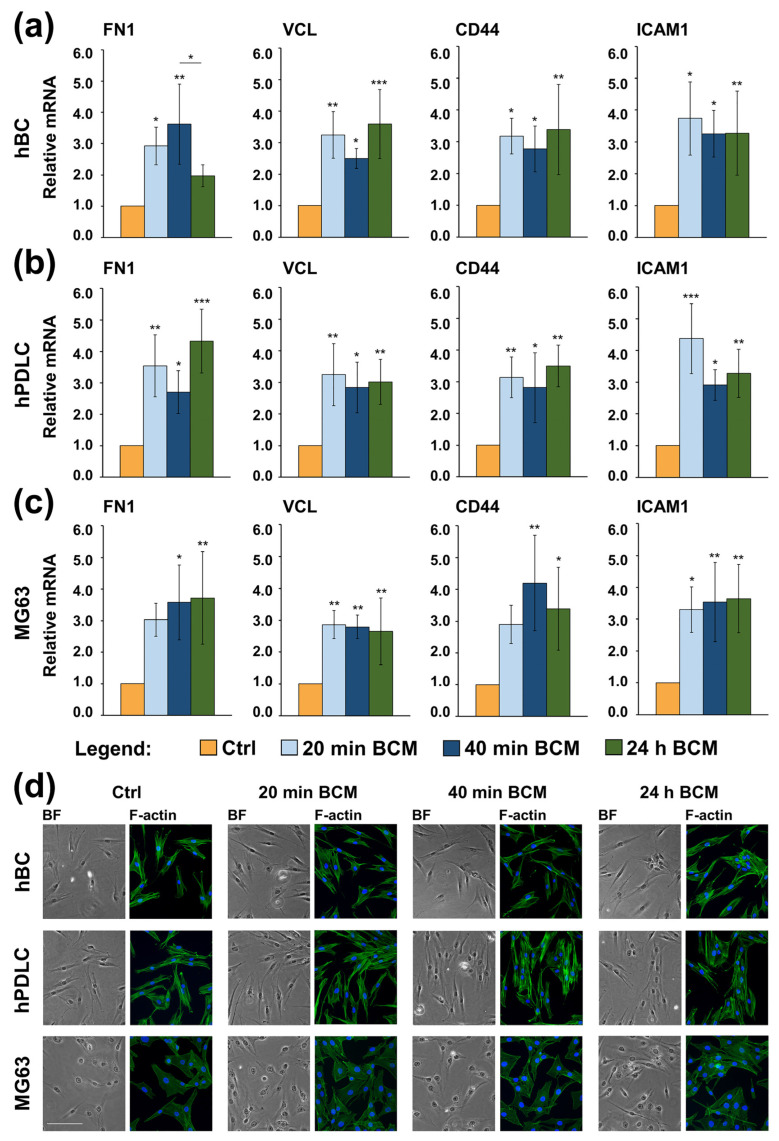
Increased expression of adhesive marker genes in hBCs (**a**), hPDLCs (**b**), and MG-63 cells (**c**) grown on BCM-free nbCM (Ctrl) or nbCMs coated with either 20 min BCM, 40 min BCM, or 24 h BCM. Cells were cultured on the respective nbCM for 6 h followed by an extensive wash for complete removal of nonadherent cells from the membranes before total RNA was isolated and analyzed for the expression of adhesive marker genes (FN1, VCL, CD44, and ICAM1) by qRT-PCR. Values normalized to GAPDH are expressed relative to the values of control cells. Data represent means ± SD from four independent experiments performed with (1) two independent BCM preparations, each used with two different cell donors for each of the primary cell types, hBC and hPDLC, and (2) four independent BCM preparations used with the MG-63 cell line. Significant differences to the respective controls, *** *p* < 0.001, ** *p* < 0.01, * *p* < 0.05. Morphological appearance and filamentous actin (F-actin) formation in hBCs, hPDLCs, and MG-63 cells, which, after initial attachment on control or BCM-coated nbCMs for 6 h, were detached and re-seeded on regular cell culture-treated plastic dishes for 24 h (**d**). Re-attached cells from all tested conditions were subjected to F-actin immunostaining using Alexa Fluor 488-labeled phalloidin (green). The cell nuclei were localized via DAPI co-stain (blue); a bright field (BF) image is also shown. Scale bar, 500 µm.

Quantification of the expression of FN1 (encoding fibronectin) [52], VCL (encoding vinculin) [53], CD44 (encoding the CD44 antigen) [54], and ICAM1 (encoding the intercellular adhesion molecule-1) [55], all playing a central role in regulating cell–cell and cell–matrix interactions, revealed a significant (*p* < 0.05) induction of all four mRNA transcripts in each of the three cell types grown on the BCM-coated nbCM compared with the expression levels detected in control cells (Figure 4a–c). The observed upregulation was in the range of 2.0–4.4-fold and appeared comparable in all three cell types. There were very few exceptions in which the BCM-coated nbCM upregulated the expression of FN1 (Figure 4a,c) and CD44 (Figure 4c) moderately but not significantly above the expression levels of the two genes in control cells.

Next, we investigated the adhesive ability and morphology of cells, which, after initial attachment on control or BCM-coated nbCMs for 6 h were detached and re-seeded on regular cell culture-treated plastic dishes for 24 h. Cells from all tested conditions were able to re-attach, and F-actin immunostaining revealed normal morphology with well-formed actin filaments (Figure 4d). Compared to control cells, and although not quantitative, the immunostaining revealed a slightly higher cell number in the test groups originating from BCM-coated nbCM, especially for hPDLC and MG-63 lines.

In summary, our data demonstrate a potent pro-adhesive capacity of the three BCM preparations coated on nbCM with no difference in their potency. Furthermore, an effect of the BCM-coated nbCM on the proliferative properties of osteoblast-like cell cultures was strongly suggested.

### 2.4. Significantly Enhanced Proliferation of Primary hBCs and hPDLCs Grown on BCM-Coated nbCM

**Figure 5:**
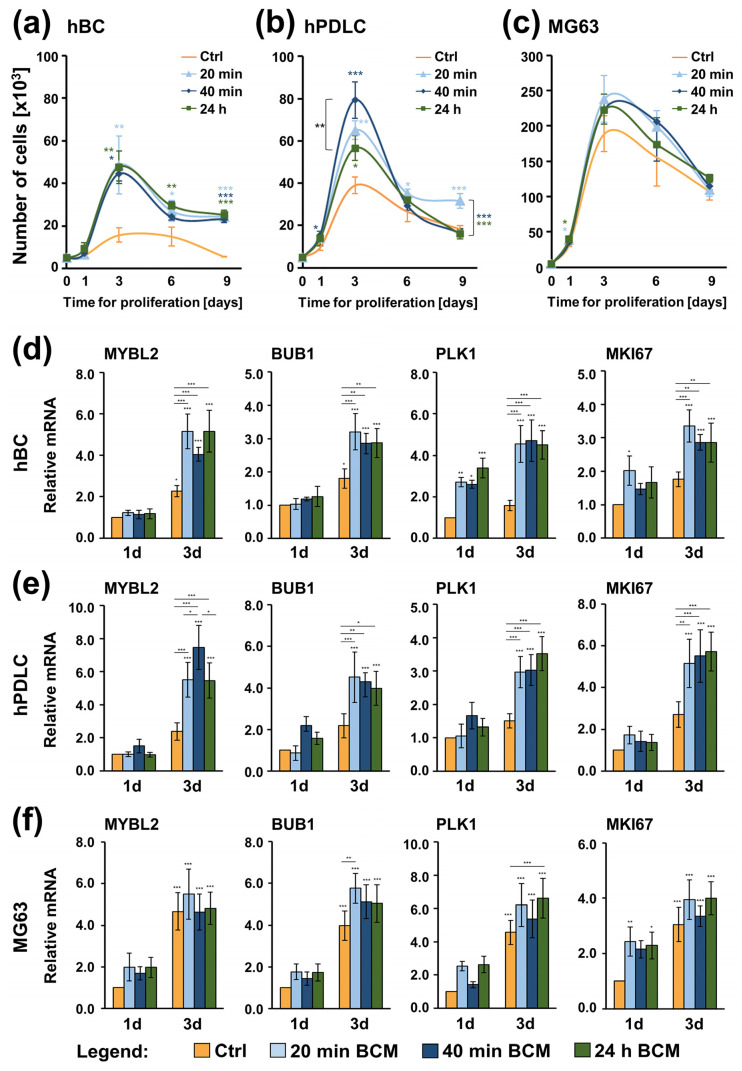
(**a**–**c**) Proliferation rates of hBCs (**a**), hPDLCs (**b**), and MG-63 cells (**c**) grown on BCM-free nbCM (Ctrl) or nbCMs coated with either 20 min, 40 min, or 24 h BCM preparation were assessed by trypan blue dye-exclusion cell counting performed in a Countess™ II instrument on days 1, 3, 6, and 9 post-seeding. Data represent means ± SD from four independent experiments performed with (1) two independent BCM preparations, each used with two different cell donors for each of the primary cell types, hBC and hPDLC, and (2) four independent BCM preparations used with the MG-63 cell line. Significant differences to control cells at each individual time point unless otherwise indicated, *** *p* < 0.001, ** *p* < 0.01, * *p* < 0.05. (**d**–**f**) Increased expression of proliferative marker genes in hBCs (**d**), hPDLCs (**e**), and MG-63 cells (**f**) grown on control or BCM-coated nbCMs. Cells were grown in the four tested conditions for 1 and 3 days before total RNA was isolated and analyzed for the expression of proliferative marker genes (MYBL2, BUB1, PLK1, and MKI67) by qRT-PCR. Values normalized to GAPDH are expressed relative to the values of control cells at day 1 (1d). Data represent means ± SD from four independent experiments performed with (1) two independent BCM preparations, each used with two different cell donors for each of the primary cell types, hBC and hPDLC, and (2) four independent BCM preparations used with the MG-63 cell line. Significant differences to the respective controls at day 1 unless otherwise indicated, *** *p* < 0.001, ** *p* < 0.01, * *p* < 0.05.

The effect of BCM-coated nbCMs on the proliferation potential of hBCs, hPDLCs, and MG-63 cells was assessed by determining the number of viable cells over a 9-day period (Figure 5a–c). All three cell types, cultured on control nbCM or nbCMs coated with each of the three BCM preparations (20 min, 40 min, or 24 h), displayed similar sigmoidal shape growth curves with a fast logarithmic growth between days 1 and 3, suggesting good biocompatibility of the nbCM. Primary hPDLCs cultured on nbCM coated with the 40 min BCM (Figure 5b), as well as MG-63 cells cultured on nbCMs coated with either 20 min or 24 h BCM (Figure 5c), showed significantly (*p* < 0.05) faster growth compared to control cells, which was already detectable on day 1. Compared to control cells, hBC cultured on BCM-coated nbCMs was the only cell type that showed consistently higher proliferative rates on days 3, 6, and 9 (Figure 5a). At the peak of growth on day 3, hPDLCs seeded on BCM-coated nbCMs showed significantly (*p* < 0.05) higher but variable growth rates compared to control cells (Figure 5b). Compared with the BCM-free nbCM, the nbCM coated with the 40 min BCM induced the most significant (*p* < 0.001) growth, which appeared significantly (*p* < 0.01) higher than the growth of hPDLCs seeded on the nbCM coated with the 24 h BCM (Figure 5b). Interestingly, no significant differences in the proliferative rates between control MG-63 and MG-63 cultured on BCM-coated nbCM were observed after day 1 due to the high proliferative capacity characterizing the control cells (Figure 5c).

The obtained results were further confirmed by investigating the expression of genes encoding proliferative markers that appear to be prominent regulators of the cell cycle progression [56] (Figure 5d–f). These are MYBL2 encoding the Myb-related protein B, BUB1 encoding a mitotic checkpoint serine/threonine-protein kinase, PLK1 encoding the polo-like kinase 1, and MKI67 encoding the Ki-67 proliferative marker. PLK1 expression in hBCs cultured on BCM-coated nbCMs (Figure 5d), MKI67 expression in hBCs cultured on nbCM coated with the 20 min BCM (Figure 5d), as well as the MKI67 expression in MG-63 line cultured on the nbCMs coated with the 20 min or 24 h BCM (Figure 5f) were the only genes whose expression was already significantly (*p* < 0.05) increased on day 1 compared to the respective controls. In agreement with the growth curve analyses, all proliferative markers tested were significantly (*p* < 0.05) induced in hBCs and hPDLCs grown on BCM-coated nbCMs for 3 days above the expression levels detected in control cells (Figure 5d,e). In agreement with the high basal proliferative potential of MG-63 cells, the expression of proliferative markers was significantly upregulated (*p* < 0.001) on day 3 above their expression levels in control cells on day 1 with almost no detectable differences between the experimental groups (Figure 5f).

Overall, BCMs harvested within short time periods and coated on nbCM for a clinically relevant 10-min period strongly stimulated the proliferation of the primary hBCs and hPDLCs but caused no further increase in the proliferative rates characterizing cancer MG-63 cells grown on BCM-free nbCMs.

### 2.5. Differential Expression of Osteogenic Marker Genes in Osteoblast-like Cell Types Grown on nbCM Coated with Short- Versus Long-Term Extracted BCM

**Figure 6:**
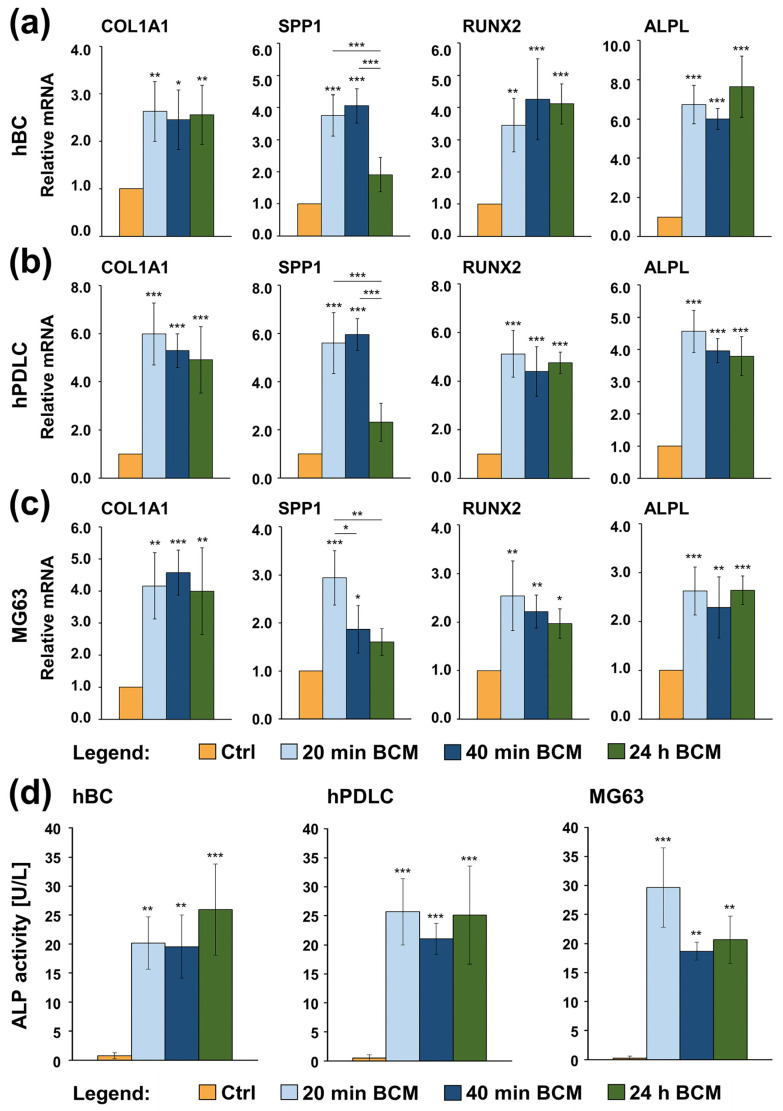
(**a**–**c**) Increased expression of early osteogenic marker genes in hBCs (**a**), hPDLCs (**b**), and MG-63 cells (**c**) grown on control or BCM-coated nbCMs. Cells were grown in the four tested conditions for 3 days before total RNA was isolated, purified, and analyzed for the expression of COL1A1, SPP1, RUNX2, and ALPL osteogenic markers by qRT-PCR. Values normalized to GAPDH are expressed relative to the values of control cells. Data represent means ± SD from four independent experiments performed with (1) two independent BCM preparations, each used with two different cell donors for each of the primary cell types, hBC and hPDLC, and (2) four independent BCM preparations used with the MG-63 cell line. Significant differences to the respective controls unless otherwise indicated, *** *p* < 0.001, ** *p* < 0.01, * *p* < 0.05. (**d**) Increased alkaline phosphatase (ALP) activity in hBCs, hPDLCs, and MG-63 cells grown on control or BCM-coated nbCMs. Cells were grown as in (**a**–**c**) before ALP activity in the cell culture supernatants was measured by a fluorometric analysis based on the hydrolysis of 4-methylumbelliferyl phosphate by the ALP into the fluorescent product 4-methylumbelliferone. Data and statistical significance are expressed as in (**a**–**c**).

**Figure 7:**
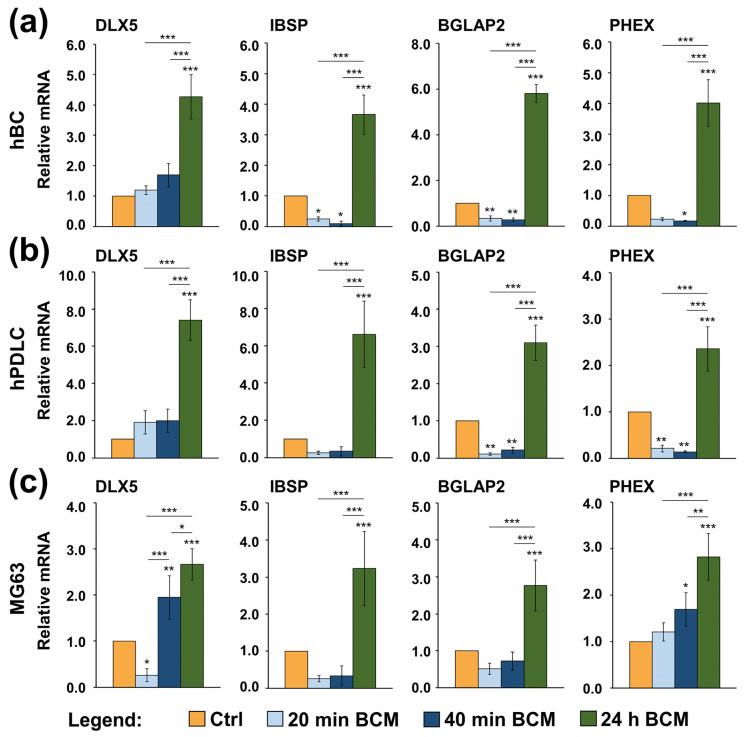
Differential expression of intermediate and late osteogenic marker genes in osteoblast-like cell types grown on nbCM coated with short- versus long-term extracted BCM. hBCs (**a**), hPDLCs (**b**), and MG-63 cells (**c**) were grown on control or BCM-coated nbCMs for 3 days before total RNA was extracted, purified, and analyzed for the expression of DLX5, IBSP, BGLAP2, and PHEX osteogenic markers by qRT-PCR. Values normalized to GAPDH are expressed relative to the values of control cells. Data represent means ±SD from four independent experiments performed with (1) two independent BCM preparations, each used with two different cell donors for each of the primary cell types, hBC and hPDLC, and (2) four independent BCM preparations used with the MG-63 cell line. Significant differences to the respective controls unless otherwise indicated, *** *p* < 0.001, ** *p* < 0.01, * *p* < 0.05.

To evaluate the osteoinductive potential acquired by the nbCM as a result of its hydration with BCM, we analyzed the expression of osteogenic marker genes in hBCs, hPDLCs, and MG-63 cells grown on the BCM-coated nbCMs (Figure 6 and Figure 7). The expression analysis included (1) genes encoding bone matrix proteins such as collagen type I (COL1A1) and secreted phosphoprotein 1 (SPP1, also known as osteopontin); (2) genes encoding early osteogenic markers such as the runt-related transcription factor 2 (RUNX2) and alkaline phosphatase (ALPL); and (3) genes encoding intermediate and late osteogenic markers such as distal-less homeobox 5 (DLX5), integrin-binding sialoprotein (IBSP), osteocalcin (BGLAP2, also known as bone gamma-carboxyglutamate protein 2), and phosphate regulating endopeptidase homolog, X-linked (PHEX).

In most cases, BCMs made within short (20 or 40 min) and long (24 h) time periods and utilized for a short 10-min coating of nbCMs caused a significant increase in COL1A1, SPP1, RUNX2, and ALPL mRNAs in hBCs (Figure 6a), hPDLCs (Figure 6b), and MG-63 cells (Figure 6c) above the expression levels detected in control cells of each type. Moreover, no significant differences in the potential of the three BCM preparations to upregulate the mRNA levels of the early osteogenic differentiation markers were evident. The only exception was the SPP1 transcript, whose expression in cells of all three types grown on nbCM coated with the 24 h BCM appeared not significantly changed compared to the respective controls (Figure 6a–c). However, it appeared significantly (*p* < 0.001) downregulated by 2.0–2.6-fold compared to its expression in hBCs and hPDLCs cultured on nbCMs coated with the 20 or 40 min BCM as well as moderately (*p* < 0.01) downregulated by 1.8-fold compared to its expression in MG-63 cells cultured on nbCM coated with the 20 min BCM. Furthermore, although slightly but significantly (*p* < 0.05) increased expression of SPP1 was observed in MG-63 cells cultured on the 40 min BCM-coated nbCM compared to the control, its expression was significantly (*p* < 0.05) decreased by 1.6-fold compared to MG-63 cells cultured on the 20 min BCM-coated nbCM (Figure 6c).

Next, we assessed the alkaline phosphatase (ALP) activity in culture supernatants of hBCs, hPDLCs, and MG-63 cells grown on the BCM-coated nbCM. In agreement with the ALPL gene expression analysis, the ALP activity in all three cell types grown on the BCM-coated nbCM was significantly (*p* <0.01) increased compared to control cells, in which the ALP activity was below the detection limit of the fluorometric analysis used (Figure 6d). The ALP activity was comparable in the cells grown on the BCM-coated nbCMs of the three osteoblast-like cell lines and was in the range of 18.7–29.6 U/L.

In contrast to the expression of the early osteogenic markers, the expression of DLX5, IBSP, BGLAP2, and PHEX appeared mostly decreased in the three osteoblast-like cell types cultured on nbCM coated with the 20 or 40 min BCM compared to control cells (Figure 7). For the late differentiation markers IBSP (Figure 7a), BGLAP2 (Figure 7a,b), and PHEX (Figure 7a,b), the downregulation was most significant, suggesting an inhibitory effect of the nbCM coated with short-term extracted BCM on the osteoblast maturation of hBCs and hPDLCs. In contrast, compared to control cells, the BMP-responsive transcriptional regulator DLX5 as well as the PHEX mRNA levels appeared slightly but significantly upregulated in MG-63 cells grown on nbCM coated with the 40 min BCM, suggesting a higher sensitivity of this cell line to the small quantities of BMP-2 present in this BCM preparation (Figure 7c). In all three cell types, the 24 h BCM coated on nbCM had a strong (*p* < 0.001) stimulatory effect on the expression of all four late osteogenic markers (Figure 7a–c).

Taken together, our results strongly suggest that nbCM adsorbs and accumulates factors contained in the BCM that can strongly potentiate the early stages of osteogenic differentiation, namely the production of extracellular matrix that will later on enable mineral deposition. Furthermore, our data suggest that BCM released from the autologous bone chips in situ, 24 h post-augmentation, may initiate the late osteogenic processes, namely osteoblast maturation, which is likely due to the presence of BMP-2 or its interaction with other factors in the complex BCM protein mixture.

## 3. Discussion

In the context of bone augmentation procedures via GBR, bilayer collagen membranes are often used to prevent the invasion of soft tissue cells while promoting the repopulation of the bone defect by osteoblast-like cells with osteogenic potential. As collagen is a major component of bone and periodontal connective tissue and the most abundant protein in the human body, it appears to be fully biocompatible [18], chemotactic for various cell types [18,57,58], degradable to nontoxic physiological compounds [59], and most importantly, able to bind to various proteins naturally present in the surrounding fluids and tissues or supplied exogenously [21]. To avoid adverse effects of exogenously administered growth factors due to non-physiological concentrations, natural sources of growth factors, in combination with suitable delivery systems, are becoming increasingly attractive [60]. In recent years, autologous platelet concentrates containing high amounts of growth factors are often applied in periodontal surgical procedures and have shown certain positive effects in bone tissue regeneration [61,62,63]. However, these regenerative approaches have not always yielded satisfactory results in terms of bone regeneration and have shown insufficient clinical predictability [64]. Therefore, we have considered BCM as a natural source of growth factors originating from bone tissue and aiming to support the regeneration of the same tissue. Previous studies have demonstrated that a 24-h extraction of porcine cortical bone chips with cell culture medium contains more than 150 proteins [35], many of which have the potential to influence cellular processes in a variety of cell types [37,38,39,40]. Moreover, we have previously reported a rapid and significant release of TGF-β1 from autogenous bone within 10 min and a specific crosstalk between TGF-β1 and BMP-2 as the mechanism by which BCM exerts its activity on the osteogenic differentiation of mesenchymal stromal cells [41]. Recently, we have also demonstrated the bio-functionalization of an osteoconductive xenogenic bone substitute material with a short-term extracted BCM [45]. However, more knowledge is needed to find ways to increase the therapeutic potential of the barrier collagen membranes by transferring the osteoinductive properties of the BCM to them. Therefore, the present study aimed at (1) characterization of the BCM composition, (2) analysis of the adsorption capacity of nbCM as a classic example of a barrier membrane, (3) analysis of the release kinetics of bioactive substances contained in the BCM from the nbCM, and (4) analysis of the cellular responses to BCM-coated nbCM. We hypothesized that the nbCM could be biofunctionalized by a clinically relevant short-term coating with a short-term extracted BCM. To test this hypothesis, we chose to use the most researched commercially available collagen membrane with a bilayer structure that enables the regulation of its barrier function [65], as well as three bone-relevant cell types. The suitability of hBCs [45], hPDLCs [66], and MG-63 [67] cells as experimental models for investigating osteoblast function has already been confirmed. While primary hBCs are true bone-derived cells, the primary hPDLCs are predominantly fibroblastic. However, the hPDLC population includes a small stem cell fraction possessing a high capacity for proliferation, self-renewal, and multilineage differentiation [68,69], making this cell type an important model for studying cellular responses toward regenerative biomaterials [70]. Considering the general assumption that sub-optimal levels of growth factors may exhibit cancerogenic activity [71], we have also included the osteosarcoma-derived MG-63 cell line in the study with the aim of revealing potential adverse effects of the proposed activation of the nbCM with BCM on bone-derived cancer cells. However, the limitation of this immortalized cancer cell line as an osteogenic model should be considered, and data should be viewed with caution. All three cell types used exhibited osteoblastic characteristics, slightly more mature for the MG-63 line.

Using the outlined tools, we produced unambiguous results that allowed us to draw important conclusions. Along with the TGF-β1 and BMP-2, whose content in BCM extracted from cortical bone chips over time has already been investigated [41], we analyzed the quantities of IGF-1, FGF-2, VEGF-A, and IL-11, all known to be stored in bone matrix and continuously released in the bone microenvironment [72]. BCM, extracted within short (20 min or 40 min) time periods corresponding to a typical surgical procedure of implant placement with simultaneous bone grafting, as well as BCM extracted within 24 h corresponding to the BCM released from the autologous bone chips in situ, contained significant and comparable quantities of TGF-β1, IGF-1, FGF-2, VEGF-A, and IL-11. In line with the previous findings [41], BMP-2 was the only growth factor present at low but significant levels in the 24 h BCM. The bioactive substances contained in all three BCM preparations were adsorbed to the nbCMs with almost 100% efficiency and released almost completely within a relatively short period of 4 days. The low levels of growth factors naturally present in the BCM, combined with the thin thickness of the membrane, justified their rapid and complete release within 4 days. Indeed, we previously observed a much slower and sustained release of recombinant growth factors loaded at much higher concentration (100 ng) from thicker 3D collagen matrices [32]. It has also been suggested that the pore size and porosity of the scaffold can significantly influence the release kinetics of bioactive factors [73]. In the present study, the growth factor release from the BCM-coated nbCM occurred from the entire bilayered surface that appears quite heterogeneous with respect to porosity [74].

We have further shown that all investigated proteins were characterized with a burst release at a relatively early time point, which, however, occurred later than the release of recombinant proteins applied to the nbCM at quantities comparable to those measured in the BCM. This result is consistent with a study showing that rBMP-2-soaked collagen sponges lacked sustained release characteristics, exhibiting high burst release and retaining less than 5% protein after a two-week period [75]. It should be noted that although the release from the BCM-coated nbCM was not sustained, it occurred sequentially for the different growth factors. The fastest release was observed for TGF-β1, IGF-1, and FGF-2, all three acting in early phases of the bone regeneration process and required for stimulating the synthesis of bone matrix and recruitment of osteoprogenitors [76,77]. The peak of the release of VEGF-A and BMP-2, which act in the intermediate and late stages of the regeneration process by stimulating neo-vascularization [78] and osteogenic differentiation and mineralization [79], respectively, was observed at 24 h. The slowest release from the BCM-coated nbCMs was detected for the IL-11, with a peak at 72 h, consistent with its function in inducing osteoclastogenesis as part of the bone remodeling process [80]. Thus, the nbCM proved to be a good delivery system for BCM and growth factors in general, as it permitted a fast but sequential release of the bioactive substances, reflecting the stages of the natural regeneration process. It is expected that the local release of multiple growth factors with the correct spatio-temporal kinetics will improve functional tissue recovery with lower doses of the loaded protein. Furthermore, since individual growth factors possess different functionalities depending on the target tissue and local microenvironment, the appropriate combination of growth factors may act cooperatively in regulating bone repair and regeneration [81]. We have previously shown that TGF-β1 and BMP-2 act synergistically for inducing osteogenic differentiation and matrix mineralization of osteoprogenitors in vitro [41]. Others have shown a similar effect of IL-11 in enhancing BMP actions in bone [82]. These findings strongly suggest that namely the combined activity of bioactive substances contained in the BCM determines its biological potential and influences the cellular responses to BCM-coated biomaterials. Our data showed that the BCM-coated nbCM was fully biocompatible for all three osteoblast-like cell types and significantly enhanced their migratory, adhesive, and proliferative phenotype. Other studies reported that the same nbCM exhibited excellent cytocompatibility for PDLCs and the osteoblast-like SaOs-2 cell line [18,83]. Interestingly, the osteosarcoma cell line MG-63 cultured on BCM-free nbCM exhibited high proliferation rates that were not further enhanced by BCM, suggesting that BCM-coated nbCM has no detrimental effects on cancer cell growth. Overall, nbCMs coated with each of the three BCM preparations caused significant induction of early osteogenic marker gene expression and induced ALP activity, suggesting an important role of the biofunctionalized nbCM in the initiation of the osteogenic process. The 24 h BCM loaded on the nbCM was the only BCM preparation that caused significant induction of late osteogenic marker genes. With high probability, this property can be attributed to the stimulatory effect of TGF-β1 and IL-11 on the BMP-2-induced osteogenesis [41,82]. The small differences in the responses of the three cell types to the BCM-coated nbCMs were most likely due to the differences in their origin and differentiation state. A study by Osyczka et al. supports this hypothesis by showing that rBMP-2 differentially stimulated bone marrow stromal cell (BMSC) differentiation depending on the age and site of BMSC harvest [84]. Thus, MG-63 cells appeared to be more sensitive to the low levels of BMP-2 present in the 40 min BCM and responded with higher expression of the BMP-responsive transcriptional regulator DLX5 when cultured on the 40 min BCM-coated nbCM. Similarly, MG-63 cells responded with higher expression of the pre-osteocyte marker PHEX and a corresponding decrease in expression of SPP1, which encodes the PHEX substrate osteopontin, compared to hBCs and hPDLCs.

Future research employing transcriptomics of RNA extracted from cells grown on BCM-coated nbCM is warranted in order to determine all genes whose expression is significantly modulated in response to short- versus long-term extracted BCM. Such investigations would shed light on the signaling pathways triggered by the BCM-activated collagen membrane. Although the use of the same BCM extraction and biomaterial coating procedures for two different types of xenogenic materials in [45] and the current study, which are often used together in GBR procedures, facilitate future standardization of the clinical protocol, any material biofunctionalization protocol resulting from our in vitro research would require animal testing. As an in vitro study, the present study cannot fully reproduce the in vivo situation, which is a clear limitation. Protein release from a collagen membrane in vivo may be very different due to the complex microenvironment in which the collagen delivery system must function. The cells attracted to the collagen compartment produce enzymes, which contribute to the degradation of collagen membranes, and this may ultimately change the speed of delivery of adsorbed proteins compared to the speed measured in vitro. Moreover, components of the tissue fluids may react with the proteins released by the autologous bone particles or compete for their binding sites with collagen.

Altogether, our data support the therapeutic potential of biofunctionalizing non-osteoinductive collagen barrier membrane with short-term extracted BCM. The BCM-coated nbCM potentiated all steps of the natural regeneration process by inducing migration of osteoblast-like cells, their adhesion, and growth on the membrane, and finally initiating their osteogenic differentiation without triggering immediate and late osteogenesis. The BCM-coated nbCM is fully biocompatible and causes no adverse effects on the growth and behavior of MG-63 cells, as a classic example of bone cancer cells. Although only partially revealed, the complex composition of the BCM, enriched in TGF-β1, IGF-1, FGF-2, VEGF-A, IL-11, and BMP-2, suggests their combinatorial action in regulating the above cellular processes. The nbCM is able to acquire the osteogenic potential of BCM and further release it in its local environment. Thus, BCM-based biofunctionalization of nbCM represents a promising therapeutic approach for routine and complex bone defects in daily clinical practice.

## 4. Materials and Methods

### 4.1. BCM Preparation and ELISA Protein Quantification

BCM was prepared as described [41,45]. In brief, cortical bone chips were harvested from the buccal side of fresh pig mandibles (Slaughterhouse: Küng Metzgerei, Toffen, Switzerland) using a bone scraper (Hu-Friedy, Rotterdam, The Netherlands) and placed into RS (Fresenius Kabi, Oberdorf NW, Switzerland) supplemented with 1% antibiotics/antimycotics (AA; ThermoFisher Scientific, Basel, Switzerland) for 20 min, 40 min, or 24 h. A ratio of 5 g bone chips per 10 mL medium was used. Based on previous studies [41,45], the 20- and 40-min BCM preparations were designated as clinically relevant short-term extractions, corresponding to the time course of a typical GBR, while the 24-h BCM preparation was designated as a long-term extraction to clarify the role of BCM released from the grafted bone particles in situ one day after the surgical treatment.

The release of TGF-β1, IGF-1, FGF-2, VEGF-1, IL-11, or BMP-2 protein in BCM preparations was quantified using Quantikine^®^ colorimetric sandwich ELISA (R&D Systems, Zug, Switzerland) according to the manufacturer’s procedure. Absorbance was measured at 450 and 570 nm on a Varioskan LUX (ThermoFisher Scientific). Data represent means ± SD from four independent experiments run in duplicates.

### 4.2. Quantification of Protein Adsorption and Release over Time to and from Collagen Membranes, Respectively

Square pieces (10 × 10 mm) of a nbCM (Geistlich Bio-Gide^®^, Geistlich, Wolhusen, Switzerland) were incubated for 10 min at room temperature with 1 mL of each of the BCM preparations or recombinant proteins, TGF-β1, IGF-1, FGF-2, VEGF-1, IL-11, or BMP-2 (all from PeproTech, London, UK) used at the average concentrations measured in the BCM preparations (as described in the Results section). The 10-min coating duration of nbCM with BCM was chosen based on (1) clinical empirical experience showing that a duration longer than 10 min would not be clinically relevant and (2) a previously published study testing coating durations from 1 min to 4 h for their efficiency in inducing a linear increase in TGF-β target gene expression in vitro [85]. As a negative control, membranes were incubated in RS alone. To remove unbound protein after the 10-min incubation, the nbCMs were extensively washed with RS for three cycles of 5 min each. Membranes were transferred in 0.5 mL RS containing 0.1% bovine serum albumin (BSA; Merck, Buchs, Switzerland) and 1% AA for incubation at 37 °C for 4 days with shaking at 70 rpm. The 0.5-mL supernatants were collected in low protein binding tubes, stored at −80 °C, and replaced with 0.5 mL of fresh RS/BSA/AA solution at 3 and 6 h, and 1, 2, 3, and 4 days. The amount of protein that remained unadsorbed to the nbCM, as well as the protein released at the indicated time points, was determined by ELISA, as described in Section 4.1. Protein adsorption by the nbCM was quantified by subtracting the amount of unbound growth factor (determined in the collected and pooled washing solution) from the total amount of protein initially added to the membranes, and was expressed in percent. The amount of released protein was calculated as a percent of the adsorbed protein. Three independent experiments with three replicates were performed for each experimental group.

### 4.3. Cell Culture

Primary hBCs and hPDLCs were obtained from three donors each by using the tissue explant techniques described previously [45,58]. Bone particles (for obtaining hBCs) derived from the retromolar area or healthy periodontal ligament (for obtaining hPDLCs) derived from the middle third portion of extracted third molars were retrieved from anonymous and systemically healthy individuals. The collection of donor tissue was approved by the Ethics Committee, Berne, Switzerland, and written informed consent was obtained from each patient (ethical code ID 2018-00661 from 13 August 2018). The osteoblastic MG-63 cells, derived from a 14-year-old male patient with osteosarcoma, were obtained from the ATCC collection (CRL-1427). All three cell types were cultured in complete Minimum Essential Medium Eagle—alpha modification (α-MEM; ThermoFisher Scientific) supplemented with 10% fetal calf serum (FCS; ThermoFisher Scientific) and 1% AA. Cells that had not undergone more than six passages were starved in 0.3% FCS/α-MEM for 24 h before culturing on nbCMs.

For cell culture experiments, nbCMs were cut sterile into 10 × 10 mm pieces and placed on the bottom of 24-well ultra-low attachment plates (Corning, NY, USA) before their coating with BCM preparations made within 20 min, 40 min, or 24 h. Cells seeded on nbCMs hydrated with RS were used as control (Ctrl) throughout the study.

For differentiation experiments followed by RNA analyses, complete media were supplemented with 50 μg/mL ascorbic acid (Invitrogen, Zug, Switzerland) and 2 mM β-glycerophosphate (Invitrogen) as described [41].

### 4.4. Transwell Migration Assay

Cell migration was assayed using transwell polycarbonate membrane inserts (6.5 mm; Greiner Bio-One, St. Gallen, Switzerland) with 8 µm pore size as described [29]. After 24 h of starvation, 5 × 10^4^ cells were plated in the top insert chamber with 200 µL serum-free α-MEM. RS-hydrated or BCM-coated nbCM was placed in the lower chamber with 800 µL serum-free medium. Cells were allowed to migrate across the filter for 22 h at 37 °C before fixation in Shandon™ Formal-Fixx™ (ThermoFisher Scientific) and stained with 0.1 mg/mL crystal violet (Merck) solution. Images of duplicate inserts were acquired on an Olympus CKX41 (Olympus Life Sciences Solution, Tokyo, Japan) equipped with a ProgResCT3 camera. Migration was quantified by using the ImageJ software (Fiji: ImageJ, with “Batteries Included”) as described [86]. Data represent means ± SD from four independent experiments performed with (1) two independent BCM preparations, each used with two different cell donors for each of the two primary cell types, hBC and hPDLC, and (2) four independent BCM preparations used with the MG-63 cell line.

### 4.5. Cell Proliferation Assay

Proliferation rates of hBCs, hPDLCs, and MG-63 cells cultured on RS-hydrated or BCM-coated nbCMs were determined by trypan blue (0.4%; ThermoFisher Scientific) dye-exclusion automated cell counting performed using the Countess II device (ThermoFisher Scientific). After 24 h of starvation, 5 × 10^3^ cells/well were cultured in 3% FCS/α-MEM either on BCM-coated nbCMs (test groups) or RS-hydrated nbCM (control group), and trypan blue stain followed by automated cell counting was performed on days 1, 3, 6, and 9 post-seeding. Data represent means ± SD from four independent experiments performed with (1) two independent BCM preparations, each used with two different cell donors for each of the two primary cell types, hBC and hPDLC, and (2) four independent BCM preparations used with the MG-63 cell line.

### 4.6. Phalloidin Stain

After being fixed in 1× Formal-Fixx (ThermoFisher Scientific) for 10 min, cells were washed and blocked in phosphate-buffered saline (PBS) containing 3% BSA (Merck) and 0.1% Triton X-100 (Merck) for 10 min. Cells were then labeled with Alexa Fluor^TM^ 488 Phalloidin (Invitrogen) at room temperature for 1 h. Cells were washed in PBS containing 0.1% Triton X-100 and co-stained with 4′,6-diamidino-2-phenylindole (DAPI; Merck) at the last washing step before being mounted in Vectashield Medium (Adipogen, Fuellinsdorf, Switzerland). Images were acquired on an Olympus BX-51 (Olympus Life Sciences Solution) equipped with the fluorescent filters U-MWIBA3 for Alexa Fluor 488 and U-MNUA2 for DAPI.

### 4.7. Quantitative Reverse Transcription-Polymerase Chain Reaction (qRT-PCR) for Gene Expression Analyses

Quantitative RT-PCR was used to investigate the expression of genes encoding (1) adhesive markers (FN1, VCL, CD44, and ICAM1), (2) proliferative markers (MYBL2, BUB1, PLK1, and MKI67), and (3) osteogenic markers (COL1A1, SPP1, RUNX2, ALPL, DLX5, IBSP, BGLAP2, and PHEX).

For analysis of adhesive marker gene expression, 6 × 10^5^ cells/well were plated in 10% FCS/α-MEM either on BCM-coated nbCMs (test groups) or RS-hydrated nbCM (control group) and allowed to adhere for 6 h. After removal of the culture media, the membranes were extensively washed three times in PBS for complete removal of nonadherent cells before RNA extraction. For analysis of proliferative and osteogenic marker gene expression, after 24 h of starvation, 2.5 × 10^5^ cells/well were plated in either 3% FCS/α-MEM (for proliferation assay) or 10% FCS/α-MEM (for osteogenic assay) as described above. Proliferative marker gene expression was analyzed on days 1 and 3 post-seeding, whereas osteogenic marker gene expression was analyzed on day 3 post-seeding.

Total RNA from cells of each experimental group was extracted using TRIzol (ThermoFisher Scientific) according to the manufacturer’s protocol. The extracted RNA was additionally purified by using the RNeasy MinElute Cleanup Kit (Qiagen, Basel, Switzerland). RNA quantified on a NanoDrop 2000c (ThermoFisher Scientific) was reverse transcribed using the High-Capacity cDNA Reverse Transcription Kit (ThermoFisher Scientific). Relative transcripts for the above-listed genes normalized to GAPDH were measured using FastStart Universal SYBR Green Master ROX (Roche, Basel, Switzerland) and the primer sequences listed in Table S1. Quantitative PCR was carried out in QuantStudio 3 (Applied Biosystems, Rotkreuz, Switzerland) using a standard thermal cycling profile. The efficiency ∆∆Ct method was used to calculate gene expression levels normalized to GAPDH values and calibrated to control values. Samples were run in duplicates. Data represent means ± SD from four independent experiments performed with (1) two independent BCM preparations, each used with two different cell donors for each of the two primary cell types, hBC and hPDLC, and (2) four independent BCM preparations used with the MG-63 cell line.

### 4.8. Alkaline Phosphatase Activity Assay

ALP activity of hBCs, hPDLCs, and MG-63 cells cultured on RS-hydrated or BCM-coated nbCMs was determined by using the ALP Assay Kit (Merck) based on the hydrolysis of 4-methylumbelliferyl phosphate by ALP present in the cell culture supernatants into the fluorescent product 4-methylumbelliferone. After 24 h of starvation, 2.5 × 10^5^ cells/well were plated in 10% FCS/α-MEM on the respective nbCMs for 3 days. ALP activity was determined according to the manufacturer’s procedure by reading fluorescence intensity on a Varioskan LUX (ThermoFisher Scientific). Samples were run in duplicates. Cell number at the time of cell culture supernatant collection, determined by the trypan blue dye-exclusion automated cell counting as described above, was used for calculating a dilution factor for each cell culture supernatant tested as compensation for differential cell number in the samples. Data represent means ± SD from four independent experiments performed with (1) two independent BCM preparations, each used with two different cell donors for each of the two primary cell types, hBC and hPDLC, and (2) four independent BCM preparations used with the MG-63 cell line.

### 4.9. Statistical Analysis

All grouped data are means ± SD. Differences between groups were assessed by one-way analysis of variance (ANOVA) with Tukey’s post hoc test using GraphPad InStat Software (GraphPad, La Jolla, CA, USA), version 3.05. Values of *p* < 0.05 were considered statistically significant.

## Data Availability

All data generated and analyzed during this study are included in this article.

## Supplementary Materials

The following supporting information can be downloaded at: https://www.mdpi.com/article/10.3390/ijms26041610/s1.
