# Supplementary material for: Biofunctionalization of Collagen Barrier Membranes with Bone-Conditioned Medium, as a Natural Source of Growth Factors, Enhances Osteoblastic Cell Behavior
Source: Int J Mol Sci. 2025 Feb 13;26(4):1610. doi: 10.3390/ijms26041610 (PMC11855076; doi:10.3390/ijms26041610)
Supplement: Supplementary file 1 [file ijms-26-01610-s001.zip › ijms-3452843-supplementary.pdf]

Supplementary Materials for:

Article

Biofunctionalization of collagen barrier membranes with bone-conditioned medium, as a natural source of growth factors, enhances osteoblastic cell behavior

Harshitha Ashoka Sreeja [1,2,a], Emilio Couso-Queiruga [2,a], Clemens Raabe [2], Vivianne Chappuis [2,b] and Maria B. Asparuhova [1, 2,b*]

[1] Laboratory of Oral Cell Biology, Dental Research Center, School of Dental Medicine, University of Bern, Freiburgstrasse 3, 3010 Bern, Switzerland; harshitha.ashokasreeja@unibe.ch; mariya.asparuhova@unibe.ch
[2] Department of Oral Surgery and Stomatology, School of Dental Medicine, University of Bern, Freiburgstrasse 7, 3010 Bern, Switzerland; emilio.couso@unibe.ch; clemens.raabe@unibe.ch; vivianne.chappuis@unibe.ch
* Correspondence: mariya.asparuhova@unibe.ch
[a] Equal contribution
[b] These authors share senior authorship

Content

Figure S1: Characterization of cell lines used in the study.

Table S1: Primer sequences used in the study.

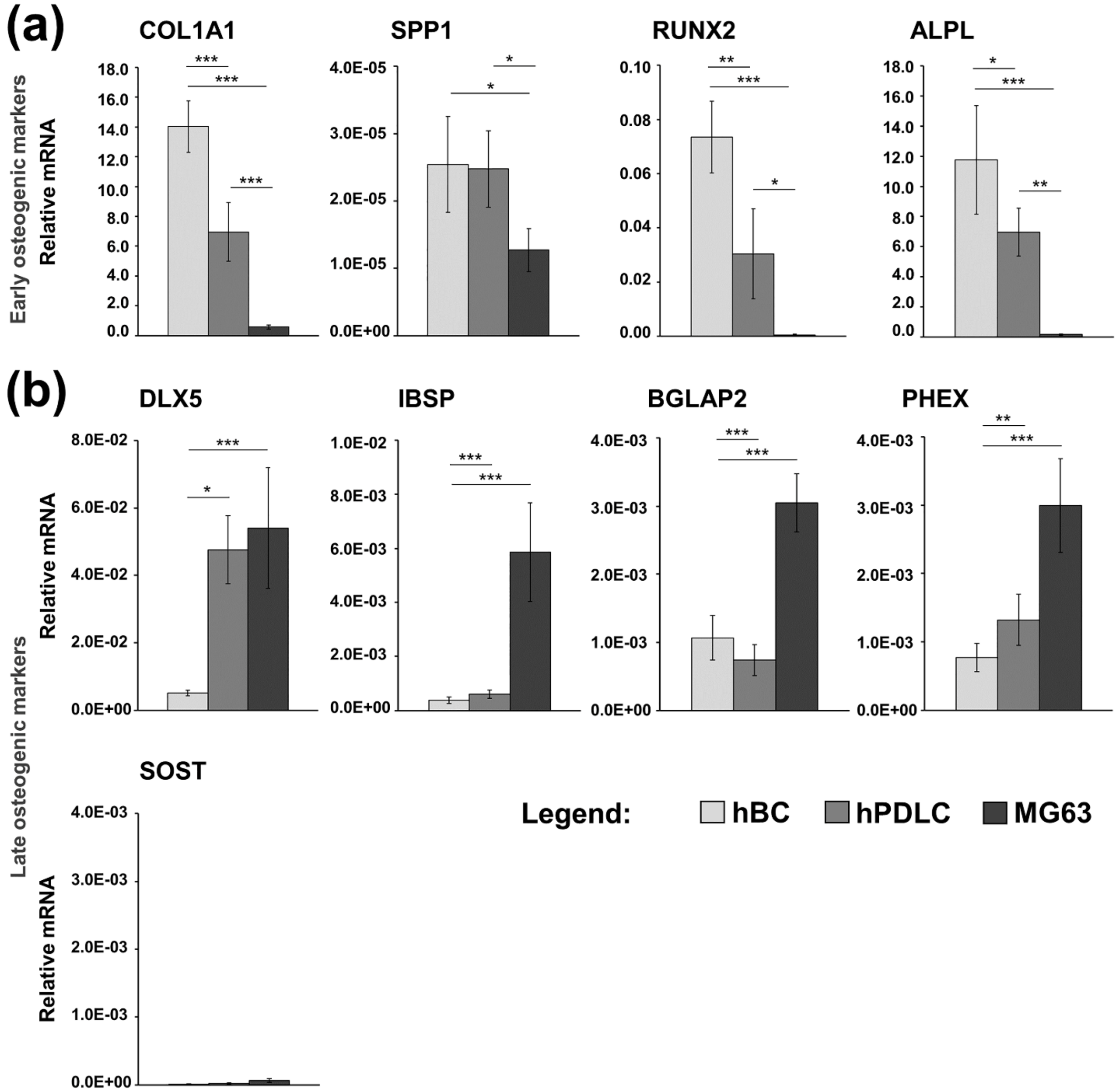

Figure S1. Characterization of cell lines used in the study. The differentiation status of primary human bone-derived cells (hBC), primary human periodontal ligament cells (hPDLC), and osteosarcoma-derived osteoblastic cell line (MG-63) was characterized by analyzing the expression of early (**a**) and late (**b**) differentiation marker genes. These are the pre-osteoblast markers (COL1A1, SPP1, RUNX2, and ALPL), osteoblast markers (DLX5, IBSP, BGLAP2, and PHEX), and the osteocyte marker (SOST). hBCs, hPDLCs, and MG-63 cells were starved in 0.3% FCS-containing medium for 24 h before total RNA was isolated and analyzed for the expression of the above-mentioned transcripts by qRT-PCR. Experimental values were normalized to GAPDH and analyzed by the ΔCt method. Data represent means ± SD from three independent experiments performed with three different donors for the primary cell lines. Significant differences ***P < 0.001, **P < 0.01, and *P < 0.05 are shown.

Table S1: Primer sequences used in the study.

Gene symbol	Primer pair (fwd/rev)
FN1	5′-CTGGCCGAAAATACATTGTAAA-3'
	5′-CCACAGTCGGGTCAGGAG-3'
VCL	5′-ATGGGTCAAGGGGCATCCT-3'
	5′-GGCCCAAGATTCTTTGTGTAAGT-3'
CD44	5′-CTGCCGCTTTGCAGGTGTA-3'
	5′-CATTGTGGGCAAGGTGCTATT-3'
ICAM1	5′-CCTTCCTCACCGTGTACTGG-3'
	5′-AGCGTAGGGTAAGGTTCTTGC-3'
MYBL2	5′-GTCAAATGGACCCATGAGGA-3'
	5′-GTCAGTGCGGTTAGGGAAGT-3'
BUB1	5′-GGAGAACGCTCTGTCAGCA-3'
	5′-TCCAAAAACTCTTCAGCATGAG-3'
PLK1	5′-AACCGAGTTATTCATCGAGACC-3'
	5′-TTGGTTGCCAGTCCAAAATC-3'
MKI67	5′-GAGGTGTGCAGAAAATCCAAA-3'
	5′-CTGTCCCTATGACTTCTGGTTGT-3'
COL1A1	5′-GAAGGGACACAGAGGTTTCAG-3'
	5′-TAGCACCATCATTTCCACGA-3'
SPP1	5′-ATGACACTGATGATTCTCACCA-3'
	5′-GCATCAGGGTACTGGATGTC-3'
RUNX2	5′-AGACCAACAGAGTCATTTAAGGC-3'
	5′-GGTGTCACTGTGCTGAAGAG-3'
ALPL	5′-TGGCAACTCTATCTTTGGTCTG-3'
	5′-TTGTTGTGAGCATAGTCCACC-3'
DLX5	5′-TTCCAAGCTCCGTTCCAGAC-3'
	5′-GAATCGGTAGCTGAAGACTCG-3'
IBSP	5′-GGAATGGCCTGTGCTTTCTC-3'
	5′-AGTCACTACTGCCCTGAACTG-3'
BGLAP2	5′-GTGCAGAGTCCAGCAAAGGT-3'
	5′-TCAGCCAACTCGTCACAGTC-3'
PHEX	5′-TTTCTTCCGGTTCGCTTGTGA-3'
	5′-AGTTCCTTCAACTTGAGGTCAAC-3'

SOST	5′-ACACAGCCTTCCGTGTAGTG-3'
	5′-GGTTCATGGTCTTGTTGTTCTCC-3'
*GAPDH	5′-ATCAAGAAGGTGGTGAAGCAG-3'
	5′-TCGTTGTCATACCAGGAAATGAG-3'

*Reference gene used for normalization in all qPCR analyses
